# *In vivo* TCR Signaling in CD4^+^ T Cells Imprints a Cell-Intrinsic, Transient Low-Motility Pattern Independent of Chemokine Receptor Expression Levels, or Microtubular Network, Integrin, and Protein Kinase C Activity

**DOI:** 10.3389/fimmu.2015.00297

**Published:** 2015-06-08

**Authors:** Markus Ackerknecht, Mark A. Hauser, Daniel F. Legler, Jens V. Stein

**Affiliations:** ^1^Theodor Kocher Institute, University of Bern, Bern, Switzerland; ^2^Biotechnology Institute Thurgau (BITg), University of Konstanz, Kreuzlingen, Switzerland

**Keywords:** CCR7, CD4^+^ T cell migration, lymph node, T cell activation, stathmin, PKC

## Abstract

Intravital imaging has revealed that T cells change their migratory behavior during physiological activation inside lymphoid tissue. Yet, it remains less well investigated how the intrinsic migratory capacity of activated T cells is regulated by chemokine receptor levels or other regulatory elements. Here, we used an adjuvant-driven inflammation model to examine how motility patterns corresponded with CCR7, CXCR4, and CXCR5 expression levels on ovalbumin-specific DO11.10 CD4^+^ T cells in draining lymph nodes. We found that while CCR7 and CXCR4 surface levels remained essentially unaltered during the first 48–72 h after activation of CD4^+^ T cells, their *in vitro* chemokinetic and directed migratory capacity to the respective ligands, CCL19, CCL21, and CXCL12, was substantially reduced during this time window. Activated T cells recovered from this temporary decrease in motility on day 6 post immunization, coinciding with increased migration to the CXCR5 ligand CXCL13. The transiently impaired CD4^+^ T cell motility pattern correlated with increased LFA-1 expression and augmented phosphorylation of the microtubule regulator Stathmin on day 3 post immunization, yet neither microtubule destabilization nor integrin blocking could reverse TCR-imprinted unresponsiveness. Furthermore, protein kinase C (PKC) inhibition did not restore chemotactic activity, ruling out PKC-mediated receptor desensitization as mechanism for reduced migration in activated T cells. Thus, we identify a cell-intrinsic, chemokine receptor level-uncoupled decrease in motility in CD4^+^ T cells shortly after activation, coinciding with clonal expansion. The transiently reduced ability to react to chemokinetic and chemotactic stimuli may contribute to the sequestering of activated CD4^+^ T cells in reactive peripheral lymph nodes, allowing for integration of costimulatory signals required for full activation.

## Introduction

Naive lymphocytes continuously home via high endothelial venules (HEVs) to secondary lymphoid organs including peripheral lymph nodes (PLNs) in search of cognate Ag (1–3). In recent years, live two-photon microscopy (2PM) imaging has uncovered that in non-reactive PLNs, naïve T cells perform random guided walk on stromal cells of the T cell area with an average speed of 10–15 μm/min (4–9). Upon encounter with cognate peptide–MHC (pMHC)-bearing DCs, T cell speeds decrease in a manner dictated by TCR affinity and the amount of pMHC complexes expressed on the surface of mature DCs. Subsequently, T cells interact continuously with one or few adjacent DCs presenting cognate pMHC, presumably through a fully matured immunological synapse (IS). Approximately 1 day later, T cells detach from DCs and regain a motile behavior, albeit with a lower speed than observed for naïve T cells (8–10 μm/min), coinciding with commitment to proliferation and the start of cytokine production (4–9). Of note, in the days following primary DC imprinting, CD4^+^ T cells remain capable to integrate secondary signals derived from late-arriving DC immigrants (10). This is in line with the observation that long-dwell times in lymphoid tissue presenting cognate pMHC complexes is required for full CD4^+^ T cell activation (11).

While the early pattern of T cell–DC interactions has been well described, such as in case of the ovalbumin (OVA)-specific DO11.10 CD4^+^ T cells (4, 6, 8), less is known about the regulation of T cell motility during the expansion phase, i.e., on days 2–6 post immunization (p.i.). In this context, chemokines have emerged as central players governing T cell motility patterns within lymphoid tissue. The CCL19/CCL21 receptor, CCR7, which is highly expressed on naïve and central memory CD4^+^ T cells, guides T cell into PLNs by activating integrins on blood-borne T cells to bind to ICAM-1 and -2 on the HEVs (12, 13). Furthermore, CCR7 ligands contribute to T cell positioning and basal scanning speed inside the T cell area (14). Upon activation and proliferation, CCR7 levels become gradually decreased, which together with CD69 expression regulates sphingosine-1-phosphate receptor 1 (S1P_1_)-mediated T cell exit from lymphoid tissue (15, 16). Subsets of activated CD4^+^ T cells increase CXCR5 expression to provide B cell help, while others increase P- and E-selectin ligand expression prior to leaving lymphoid tissue to accumulate at sites of inflammation (17–19). In addition, blood-borne naïve T cells also express CXCR4, which participates in lymphocyte adhesion in HEV (20), but does apparently not contribute to parenchymal T cell migration (21, 22). Despite these insights, it remains currently unknown how chemokine receptor levels correspond to the motility patterns observed during physiological T cell activation in 2PM experiments. Thus, decreased CD4^+^ T cell motility after DC activation may be a direct consequence of activation-induced lower expression of CCR7 or other chemokine receptors (16). Alternatively, or in addition, decreased CD4^+^ T cell motility may result from increased adhesiveness to stromal or hematopoietic cells via increased LFA-1 expression (23), and/or formation of late intercellular contacts with newly incoming DCs (15). Of note, TCR signaling may also alter intracellular signaling networks that cell-intrinsically modulate T cell motility (24). In this context, a recent report found reduced levels of phosphorylated ezrin/radixin/moesin (pERM) and increased levels of phosphorylated stathmin (pStathmin) after *in vitro* CD3-stimulated activation of human T cells (25). While lower ERM phosphorylation impairs uropod formation, increased pStathmin levels cause microtubule network stabilization that correlated with decreased *in vitro* chemotaxis (25). Whether such a mechanism correlates with migration parameters during physiological T cell activation has not been addressed to date. Interestingly, chemokine receptors also undergo regulatory processes by receptor desensitization that is initiated by the phosphorylation of the receptor upon ligand binding. In the case of CCR7, receptor phosphorylation of serine and threonine residues within the cytoplasmic loops and the C-terminus has been described to depend on G protein coupled receptor kinases (GRKs) (26) or second-messenger-dependent protein kinases including protein kinase C (PKC) (27). Notably, TCR signaling leads to activation of PKC isoforms that have been described to phosphorylate chemokine receptors in the absence of chemokine ligands to desensitize chemokine receptors in an heterologous manner (28).

In the present study, we examined motility patterns of *in vivo-*activated adoptively transferred CD4^+^ T cells using a reductionist *in vitro* chemotaxis system that allowed to precisely compare chemokine receptor surface levels with migratory capacity while employing non-TCR transgenic endogenous CD4^+^ T cells population as internal control for the inflammatory milieu. Our data uncover a cell-intrinsic loss of motility in CD4^+^ T cells shortly after activation coinciding with clonal expansion that is independent of chemokine receptor levels, microtubular network integrity, or PKC signaling. The reduced ability of CD4^+^ T cells to react to chemokinetic and chemotactic stimuli may contribute to control their lymphoid tissue dwell time, allowing subsets of activated cells integrating additional signals required for full activation before egress.

## Materials and Methods

### Reagents

Biotinylated or PE-, PerCP,- or APC-conjugated mAbs against mouse CXCR4 (clone 2B11), CXCR5 (2G8), CD44 (IM7), LFA-1 (2D7), CD25 (PC61), IL-2 (JES6-5H4), IFN-γ (XMG1.2), and PE-or APC-conjugated streptavidin were from BD Biosciences (Allschwil, CH), and FITC-conjugated anti-CD4 mAb (RM4–5) was from Biolegend (San Diego, CA, USA). CCR7 was detected using a CCL19–Ig fusion protein as described (29) (kindly provided by U. H. von Andrian, Harvard Medical School), followed by biotinylated or PE-conjugated goat anti-human Fc Abs (Beckman Coulter, Fullerton, CA, USA). The specificity of CCL19–Ig binding to CCR7 on T cells was confirmed comparing labeling of wild type and CCR7^−/−^ T cells (not shown) (29–31). Alternatively, we labeled cells with biotinylated anti-CCR7 mAb (4B12) from eBioscience, using isotype-matched biotinylated anti-rat IgG_2a_ (R35–95) as control. Unconjugated mAb for phosphorylated ezrin/radixin/meoosin (pERM) and pAb for phosphorylated Stathmin (pStathmin) were purchased from Cell Signaling Technology (#3149, Danvers, MA, USA) and Bioss (bs-9765, Woburn, MA, USA), respectively. For detection of the DO11.10-TCR specific for OVA 323–339, we employed FITC-, PE, or TRI-COLOR-conjugated mAb KJ1–26 (Caltag, Burlingame, CA, USA). Blocking mAbs against αL- (FD441.8) and α4-integrin (PS/2) were from nanotools (Freiburg, Germany). We obtained 5-(and-6)-carboxyfluorescein diacetate, succinimidyl ester [5(6)-CFDA-SE] from Molecular Probes (Eugene, OR, USA). Murine CCL19, CCL21, and CXCL12 were from Peprotech (London, UK), and murine CXCL13 was from R&D systems (Minneapolis, MN, USA). OVA, saponin, and nocodazole were purchased from Sigma (St. Louis, MO, USA). OVA peptide 323–339 (OVA_323–339_) was produced by Fernando Roncal at the National Center for Biotechnology (Madrid, Spain).

### Mice, lymphocyte preparation, and adoptive transfer

All mice used in this study were on a BALB/c background and housed and bred under specific pathogen-free conditions. Total lymphocytes were isolated from spleens and lymph nodes of 6- to 10-week-old DO11.10-TCR transgenic mice (Jackson Laboratories, Bar Harbor, ME, USA). After erythrocyte lysis, CD4^+^ T cells were isolated using negative magnetic bead separation (Dynal, Oslo, Norway, or StemCell Technologies, Grenoble, France). Purity of sorted CD4^+^ T cells was routinely >95% and contained 70–80% KJ1–26^+^ cells. Approximately, 2.5 × 10^6^ DO11.10 CD4^+^ T cells were injected i.v. into age- and sex-matched BALB/c recipient mice. In some experiments, sorted CD4^+^ T cells were labeled prior to adoptive transfer with 2 μM 5(6)-CFDA-SE for 45 min, at 37°C. One day after adoptive transfer, recipient mice received s.c. injections of a 1:1 emulsion of complete Freund’s adjuvant (CFA) and 20 mg/ml OVA (in PBS) or, alternatively, 3 mg/ml OVA_323–339_ in five points closed to inguinal, axillary, brachial, and cervical PLNs (50 μl/site). Animal work has been approved by the Cantonal Committee for Animal Experimentation and conducted according to federal and cantonal guidelines.

### Flow cytometry

At indicated times, single cell suspensions were prepared from draining PLNs (brachial, cervical, axillary, inguinal) of recipient mice as described (7), and incubated for 1 h at 37°C in complete medium (CM-R; RPMI/10% FCS/standard supplements). After blocking Fc receptors with PBS containing 5% rat and mouse serum (30 min, 4°C), cells were washed in PBS and mAbs were added. Flow cytometric analysis was carried out on an LSR II or FACScalibur and analyzed with CellQuest Pro (BD Biosciences) or FlowJo (FlowJo, Ashland, OR, USA) software. For pERM and pStathmin staining, single cell suspensions were fixed in 4% PFA/PBS immediately after harvest. Cell surface marker staining was performed as described above prior to cell permeabilization with 0.1% saponin/PBS (15 min on ice). After Fc receptor blocking, primary and secondary antibodies were incubated for 1 h and 30 min, respectively (4°C). Lymphocyte gating was performed according to FSC/SSC properties without exclusion of doublets (Figure S1 in Supplementary Material).

### Chemotaxis assays

Unsorted single cell suspensions from draining PLNs were resuspended in CM-R (5 × 10^6^ cells/ml) and chemotaxis assays were performed using 5 × 10^5^ cells/well according to the manufacturer’s instructions (Transwell 5 μm pore size, CoStar, Cambridge, MA, USA). In some experiments, cells were pretreated with indicated concentrations of Nocodazole for 10 min and throughout the chemotaxis assay. Blocking mAbs to αL- and α4-integrins were applied at 1 μg/10^6^ cells for 20 min prior to chemotaxis assays. Adhesion and homing experiments have shown complete blocking of these integrins at indicated concentrations (13). After 2 h at 37°C, 5% CO_2_, input, and migrated cells were labeled with mAbs against CD4 and DO11.10. From the number of migrated cells and the percentage of CD4^+^ and KJ1–26^+^ cells in top and bottom chamber, we calculated the percentage of migrated cells for each condition. In each experiment, we also determined spontaneous migration in the absence of chemokine, which was used to subtract chemokinesis from chemokine-induced migration. To determine cell death, we labeled input lymphocyte populations with propidium iodide at the end of the experiment. For chemotaxis of *in vitro* activated T cells, we treated lymphocytes for 2 days with anti-CD3 and anti-CD28 mAbs (1 μg/ml), washed, and mixed them with freshly isolated lymphocytes in a 1:20 ratio to mimic lymphocyte suspensions isolated from inflamed PLNs. For chemotaxis of activated T cells in the presence of PKC inhibitors, we treated splenocytes for 2 days with anti-CD3 and anti-CD28 mAbs (1 μg/ml), washed them, and treated cells with 1 or 10 μM of the pan-PKC inhibitor BIM (Bisindolylmaleimide X hydrochloride, Sigma-Aldrich, B3931, in DMSO) or DMSO for 2 h at 37°C before chemotaxis toward 25 or 50 nM CCL21 in the presence of the inhibitors or vehicle (1 × 10^5^ cells/well). The remaining activated splenocytes were used for western blot analysis as described below.

### Homing assays and 3-DIF

CD4^+^ T cells were purified from draining lymph nodes on day 3 after OVA/CFA immunization by negative selection according to manufacturer’s instructions (StemCell Technologies, Vancouver, CA, USA) and sorted for KJ1–26^+^ and KJ1–26^−^ cells using a FACS Aria III (BD Biosciences). 3.5 to 4 × 10^5^ KJ1–26^+^ and 2.4 to 3 × 10^6^ KJ1–26^−^ CD4^+^ T cells were fluorescently labeled and injected into age and sex-matched Balb/c recipients. After 3–5 h, PLNs were harvested and processed for 3-D immunofluorescence (3-DIF) as described (15). In brief, PLNs were fixed o.n. in 1% PFA/PBS, carefully cleaned from surrounding fat tissue, embedded in 1.3% low-melting point agarose (Invitrogen), dehydrated o.n. in 100% MeOH, and cleared o.n. in benzyl alcohole:benzyl benzoate (BABB, 1:2). Image stacks of 400 μm × 400 μm × 300–400 μm were imaged using a two-photon microscope (Trimscope, LaVision Biotech, Bielefeld, Germany). Cells were localized and quantified using Volocity (Improvision, Perkin Elmer, Cambridge, UK).

### Immunofluorescence staining

Naive CD4^+^ T cells from DO11.10 mice were treated for 10 min with indicated nocodazole concentrations, transferred to poly-l-lysine-coated cover slips for 20 min before fixation with 4% PFA/PBS (20 min at RT), and permeabilized using cytoperm/cytofix solution (20 min, RT; BD Biosciences). As primary antibody, we used rabbit-anti α/β-tubulin (1:50 dilution, 2 h at RT; Cell Signaling Technology #2148), followed by Alexa488-conjugated goat-anti-rabbit-IgG (1 h at RT; Invitrogen #A-11008). Samples were analyzed using a Nikon Eclipse E600 microscope equipped with a 60× oil objective (NA 1.4) and NIS elements software (Nikon).

### Western blot analysis of PKC isoforms

Freshly isolated or *in vitro*-activated splenocytes (2 × 10^6^ cells/sample) were lysed in NP40 lysis buffer supplemented with phosphatase inhibitor cocktail (PhosStop, Roche), Protease inhibitor cocktail (complete Mini, Roche), 1 mM Na_3_VO_4_, and 1 mM NaF. After determining the protein concentration, we loaded 30 μg protein/lane. All PKC mAbs used were from Cell Signaling Technology (# 2051, 2052, 2054, 9371, 9374, 9375, 9376, 9377, 9378), and anti-beta actin was from Abcam (# ab8227).

### Statistical analysis and graphs

Box and whiskers graphs were compiled using Prism (Graphpad), with the box showing the 25th to 75th percentiles and the whiskers reaching to the lowest and highest values. The line in the box shows the median. Statistical tests were performed using ANOVA or Student’s *t*-test as indicated in the figures (Prism, Graphpad). Significance was set at *p* < 0.05.

## Results

### Kinetic analysis of expansion and activation marker expression of OVA-specific DO11.10 CD4^+^ T cells during inflammation

**Figure 1:**
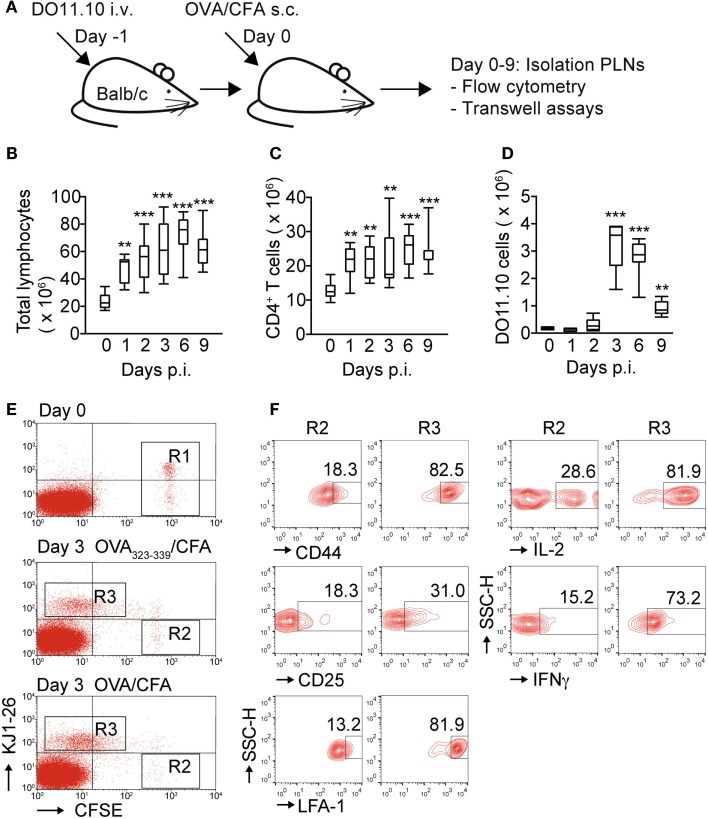
**Kinetic of DO11.10 CD4^+^ T cell expansion and activation after OVA/CFA immunization**. **(A)** Outline of experiment. DO11.10 CD4^+^ T cells were adoptively transferred 1 day before s.c. OVA/CFA immunization. Draining PLNs were analyzed by flow cytometry and chemotaxis. **(B)** Total pooled PLN lymphocyte numbers during OVA/CFA immunization. **(C)** Total CD4^+^ T cell numbers in pooled PLNs during OVA/CFA immunization. **(D)** Total KJ1–26^+^ CD4^+^ T cell numbers in pooled PLNs during OVA/CFA immunization. **(E)** Representative flow cytometry plots of adoptively transferred CFSE-labeled KJ1–26^−^ and KJ1–26^+^ cells on day 0 (top panel; R1) and on day 3 post OVA peptide/CFA (middle panel) or OVA/CFA (bottom panel) immunization (R2 and R3, respectively). **(F)** Flow cytometry analysis of activation markers of KJ1–26^−^ and KJ1–26^+^ cells in gates R2 and R3 as shown in **(E)** after priming with peptide. Similar results were obtained with OVA immunization. Numbers indicate percentages in gated populations. Data in **(B–D)** are pooled from 6 independent experiments combining 4–13 mice/time point and are shown as box and whiskers graphs as described in the section “Materials and Methods.” Flow cytometry plots in **(E,F)** are representative of two independent experiments. Statistical analysis was performed using ANOVA test against “Day 0” values followed by Dunnett’s multiple comparison test. **p < 0.01; ***p < 0.001.

Previous 2PM studies of reactive PLNs have shown that DO11.10 CD4^+^ T cells decreased their migration speed after activation by DCs, often moving slowly in spatially confined “swarms” (4, 6). To investigate the relationship between *in vivo* activation phenotype and migratory capacity to defined chemokines, we set up an inflammation model to follow the expansion of Ag-specific CD4^+^ T cells and to compare their behavior with an endogenous CD4^+^ T cell control population. To mimic experimental conditions used in previous 2PM studies, we transferred 2 to 3 × 10^6^ DO11.10 CD4^+^ T cells into syngeneic Balb/c recipient mice, which were s.c. immunized 1 day later with OVA emulsified in CFA. On the day of OVA/CFA administration, some mice did not receive OVA/CFA (=day 0), while others were sacrificed 1–9 days post immunization (p.i.) to isolate reactive PLNs for flow cytometry and chemotaxis assays (Figure 1A). While DO11.10 CD4^+^ T cell expansion is a well-known consequence of OVA immunization, we set out to determine the precise kinetics of this process, which may vary between laboratories. OVA/CFA immunization caused an increase in total PLN cell numbers, rising from 23.8 ± 5.1 × 10^6^ (mean ± SD) on day 0 to 72.1 ± 14.4 × 10^6^ cells on day 6 p.i. before slowly decreasing to 62.1 ± 12.7 × 10^6^ on day 9 p.i. (Figure 1B). Similarly, the endogenous KJ1–26^−^ CD4^+^ T cell population in reactive PLNs doubled from 12.9 ± 2.3 × 10^6^ cells on day 0 to 25.1 ± 5.0 × 10^6^ cells on day 6 p.i., and remained stable at 24.0 ± 5.1 × 10^6^ cells on day 9 p.i. (Figure 1C). In contrast to the sustained increase of endogenous CD4^+^ T cells, Ag-specific DO11.10 CD4^+^ T cells showed a much more pronounced expansion and contraction. The number of DO11.10 CD4^+^ T cells increased from 0.17 ± 0.0 × 10^6^ cells on day 0 to 3.2 ± 0.9 × 10^6^ cells on day 3 p.i. before sharply falling to 0.9 ± 0.3 × 10^6^ cells on day 9 p.i. (Figure 1D) and becoming barely detectable on day 13 p.i. (not shown). On days 4 and 5 p.i., the total numbers of PLN cells, endogenous CD4^+^ T cells, and DO11.10 CD4^+^ T cells remained on the levels of days 3–6 (not shown) indicating that after 3 days p.i., near-maximal Ag-specific CD4^+^ T cell expansion is reached. Accordingly, on day 3 p.i., we found that adoptively transferred KJ1–26^+^ but not KJ1–26^−^ CD4^+^ T cells had undergone extensive cell divisions (Figure 1E). In contrast to non-TCR transgenic KJ1–26^−^ DO11.10 CD4^+^ T cells, KJ1–26^+^ DO11.10 CD4^+^ T cells expressed proinflammatory cytokines (IL-2, IFNγ) and showed increased CD25, CD44, and LFA-1 surface levels corresponding to *bona fide* hallmarks of activation (Figure 1F).

### Dynamic regulation of CCR7, CXCR4, and CXCR5 levels on activated DO11.10 CD4^+^ T cells

**Figure 2:**
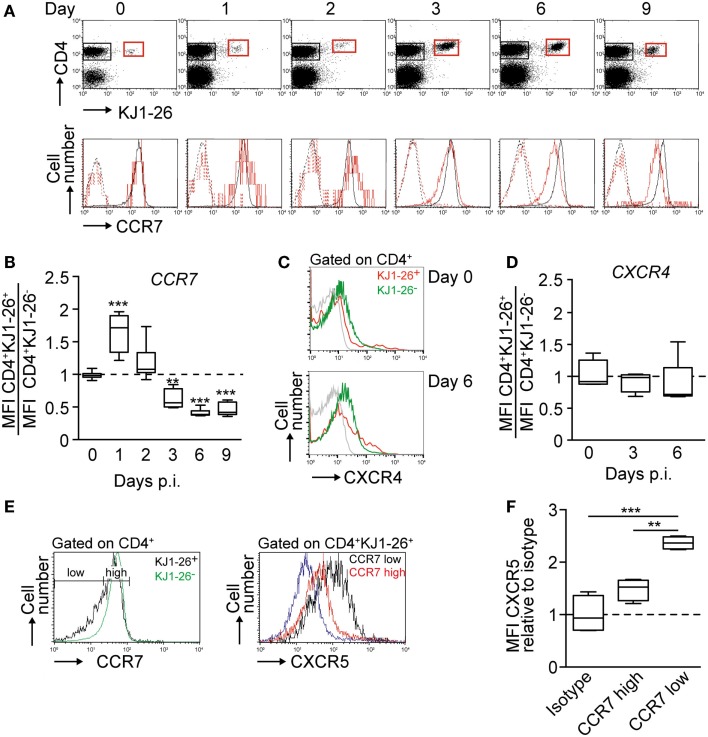
**Chemokine receptor regulation on DO11.10 CD4^+^ T cells after OVA/CFA immunization**. **(A)** Top panel. Representative flow cytometry plots showing expansion and contraction of adoptively transferred KJ1–26^+^ DO11.10 CD4^+^ T cells in OVA/CFA-draining PLNs. Bottom panel. Corresponding flow cytometry plots of CCR7 expression on endogenous CD4^+^ (black) and DO11.10 CD4^+^ T cells (red). Dashed lines show secondary mAb only. **(B)** Ratio of CCR7 MFI on KJ1–26^+^ CD4^+^ versus endogenous CD4^+^ T cells after OVA/CFA immunization. **(C)** Flow cytometry plots of CXCR4 expression on KJ1–26^+^ CD4^+^ and endogenous CD4^+^ T cells after OVA/CFA immunization. The isotype control is shown in gray. **(D)** Ratio of CXCR4 MFI on KJ1–26^+^ CD4^+^ versus endogenous CD4^+^ T cells after OVA/CFA immunization. **(E)** Left panel. Gating strategy for CCR7^low^ and CCR7^high^ CD4^+^ KJ1–26^+^ T cells on day 5 post OVA/CFA immunization. CCR7 levels on endogenous CD4^+^ KJ1–26^−^ T cells are depicted in green. Right panel. CXCR5 expression levels on CCR7^low^ (black) and CCR7^high^ (red) CD4^+^ KJ1–26^+^ T cells. The isotype control is shown in blue. **(F)** Ratio of CXCR5 MFI on CCR7^low^ and CCR7^high^ KJ1–26^+^ CD4^+^ T cells on days 5/6 after OVA/CFA immunization, normalized to isotype control. Data are pooled from 2–5 independent experiments combining 4–10 mice/time point. Data in **(B,D,F)** were analyzed using ANOVA and Dunnett’s **(B,D)** or Tukey’s **(F)** multiple comparison test and are shown as box and whiskers graphs. **p < 0.01; ***p < 0.001.

Building on the kinetics established for the OVA/CFA immunization model, we performed a side-by-side flow cytometric analysis of chemokine receptors, CCR7, CXCR4, and CXCR5, expressed on endogenous and KJ1–26^+^ DO11.10 CD4^+^ T cells. Using a CCL19–Ig fusion protein to label CCR7 (29), we found CCR7 surface expression levels on DO11.10 CD4^+^ T cells to remain similar or even slightly increased compared to endogenous CD4^+^ T cells on day 1 and 2 p.i. before gradually decreasing to 40% of levels of non-activated CD4^+^ T cells on day 9 p.i. (Figures 2A,B; Figures S1A,B in Supplementary Material). Similar results were obtained when we used an anti-CCR7 mAb (not shown). By contrast, CXCR4 MFI levels remained stable on endogenous and DO11.10 CD4^+^ cells (Figures 2C,D). Similarly, the percentages of CXCR4^+^ KJ1–26^+^ versus endogenous CD4^+^ T cells was comparable on day 3 (24.9 ± 4.5 versus 25.5 ± 1.9%, respectively) and day 6 p.i. (25.9 ± 14.0 versus 22.1 ± 3.6%, respectively). As predicted from previous studies (18), CXCR5 expression started to increase on day 3 p.i. (not shown), with an inverse correlation between CCR7 and CXCR5 levels on days 5 and 6 p.i. Thus, while 25.8 ± 1.4% of all DO11.10 CD4^+^ T cells expressed CXCR5 on days 5 and 6 p.i., this percentage increased to 48.9 ± 2.1% for the CCR7^low^ subpopulation, whereas only 14.1 ± 2.1% of CCR7^high^ DO11.10 CD4^+^ T cells expressed CXCR5 (Figure 2E). In addition, the relative CXCR5 MFI of CCR7^low^ DO11.10 CD4^+^ T cells was increased by 62% over the one of CCR7^high^ cells when normalized to isotype control (Figure 2F). At this time point, the percentage of endogenous CXCR5^+^ CD4^+^ T cells remained low at 8.1 ± 0.5%, presumably owing to strong competition by high numbers of adoptively transferred Ag-specific CD4^+^ T cells. In sum, our data uncover a gradual decrease in CCR7 levels on DO11.10 CD4^+^ T cells starting on day 3 p.i., which coincided with massive expansion and CXCR5 upregulation on a subset of transferred cells.

### *In Vitro* migration assays identify an activation-induced transient decrease of intrinsic DO11.10 CD4^+^ T cell motility uncoupled of chemokine receptor surface levels

**Figure 3:**
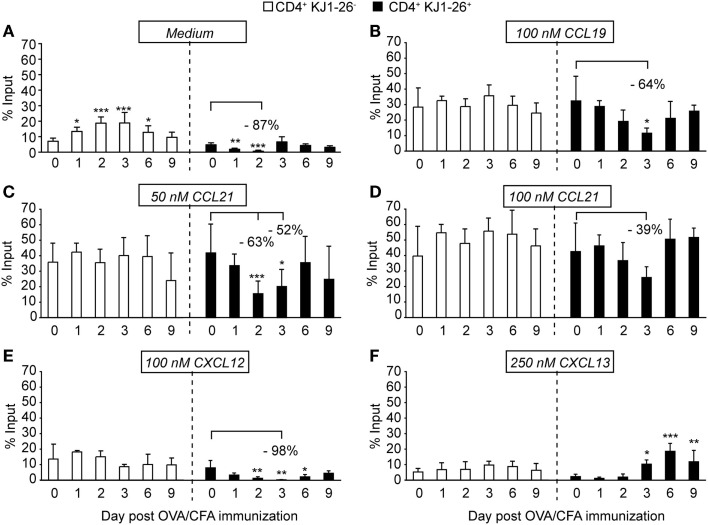
***In vitro* migration of endogenous and DO11.10 CD4^+^ T cells during OVA/CFA immunization**. **(A)** *In vitro* chemokinesis of endogenous CD4^+^ T cells (white bars) and KJ1–26^+^ DO11.10 CD4^+^ T cells (black bars) in the absence of chemokine after OVA/CFA immunization. **(B)** Chemotaxis to 100 nM CCL19. **(C)** Chemotaxis to 50 nM CCL21. **(D)** Chemotaxis to 100 nM CCL21. **(E)** Chemotaxis to 100 nM CXCL12. **(F)** Chemotaxis to 250 nM CXCL13. Chemokinetic migration **(A)** was subtracted in **(B–F)** to focus on chemokine-induced motility. Bars represent mean ± SD of % input and were pooled from three to four independent experiments combining three to eight mice per time point. Statistical analysis was performed separately for endogenous CD4^+^ T cells and KJ1–26^+^ DO11.10 CD4^+^ T cells using ANOVA test against “Day 0” values (=control migration) followed by Dunnett’s multiple comparison test. *p < 0.05; **p < 0.01; ***p < 0.001.

While parenchymal T cell migration follows a random guided motility pattern, chemokine receptors participate in their motility. We therefore performed Transwell chemotaxis assays as surrogate tool to examine the functionality of CCR7, CXCR4, and CXCR5 on activated CD4^+^ T cells during directed *in vitro* migration, while correlating these data to receptor surface levels. When we analyzed spontaneous motility of isolated endogenous and adoptively transferred CD4^+^ T cells, we observed on days 1 and 2 p.i. a transient decrease in DO11.10 CD4^+^ T cells chemokine-independent motility. By contrast, endogenous CD4^+^ T cells showed sustained chemokinetic motility peaking on day 2 and 3 p.i. (Figure 3A; Figure S1C in Supplementary Material), potentially induced by exposure to chemokinetic factors such as thromboxane A2 (32, 33). We next focused on chemokine-induced motility after subtracting the corresponding values of spontaneous migration observed in Figure 3A. We did not detect significant differences in the capacity of endogenous CD4^+^ T cells to migrate to CCL19, CCL21, CXCL12, and CXCL13 irrespective of the day p.i. analyzed, indicating that the inflammatory milieu had no effect on chemokine-induced migration of these cells (Figures 3B–F). By contrast, we observed a reduction of more than 50% in chemokine-induced migration of DO11.10 CD4^+^ T cells to 100 nM CCL19 and 50 nM CCL21 on days 2 and 3 p.i. (Figures 3B,C). DO11.10 CD4^+^ T cells showed a non-significant tendency to lower migration to 100 nM CCL21 on day 3 p.i. (Figure 3D), suggesting that decreased motility could be partially rescued using higher chemoattractant concentrations. It is noticeable that DO11.10 CD4^+^ T cell migration to CCL19 and CCL21 on days 6 and 9 p.i. returned to the levels of endogenous CD4^+^ T cells despite decreased CCR7 surface expression levels at these time points (Figure 2B). Similar to observations made with CCR7 ligands, DO11.10 CD4^+^ T cells migration to CXCL12 was virtually abolished on day 3 p.i. (Figure 3E). On the other hand, CXCL13-induced motility correlated well with increased CXCR5 expression levels on these cells during the course of the immune response (Figure 3F). Migration patterns were comparable when we quantified chemotaxis without subtraction of background migration (Figure S2 in Supplementary Material). Finally, we examined *in vitro* chemotaxis of sorted KJ1–26^+^ and KJ1–26^−^ CD4^+^ T cells on day 3 p.i., confirming a cell-intrinsic reduction in spontaneous and CCL21-induced migration of recently activated T cells in purified populations (Figure S3E in Supplementary Material). Less than 2.5% of endogenous or activated T cells were propidium iodide-positive after *in vitro* incubation for 2 h, excluding cell death as reason for decreased numbers of migrated T cells (Figure S1D in Supplementary Material). Taken together, our chemokinetic and chemotactic migration experiments uncovered a transient TCR-induced decrease of DO11.10 CD4^+^ T cell motility on days 2 and 3 p.i., which was at least partially uncoupled from CCR7 and CXCR4 expression levels.

### Reduced *In Vitro* motility of activated DO11.10 CD4^+^ T cells correlates with decreased *In Vivo* homing

To assess whether lower *in vitro* chemotaxis of activated T cells was reflected in altered lymphoid tissue motility, we performed 2PM experiments with sorted KJ1–26^−^ and KJ1–26^+^ CD4^+^ T cells on day 3 post OVA/CFA immunization, the earliest time point at which Ag-specific DO11.10 T cells had sufficiently expanded for sorting (Figure 1D). Since we still obtained too few KJ1–26^+^ CD4^+^ T cells for 2PM imaging after systemic transfer, we used microinjection to deliver fluorescently labeled T cells directly into surgically exposed popliteal PLNs prior to imaging. While we readily identified transferred T cells in lymphoid tissue by this approach, we found that their motility was severely compromised irrespective of their activation status, precluding a meaningful 2PM analysis (not shown). We also examined whether the reduced responsiveness of recently activated DO11.10 CD4^+^ T cells toward CCR7 ligands was reflected in short-term homing assays, since this receptor is together with L-selectin and LFA-1 critical for T cell entry into PLNs (2). We sorted KJ1–26^+^ and KJ1–26^−^ CD4^+^ T cells from reactive PLNs of Balb/c mice after their expansion on 3 days p.i., labeled these cells fluorescently and adoptively transferred them into non-immunized sex-matched recipient mice for 3–5 h before PLN excision (Figures S3A,B in Supplementary Material). Owing to the low number of KJ1–26^+^ CD4^+^ T cells recovered after sorting for adoptive transfer, we performed a high-sensitivity three-dimensional immunofluorescence (3-DIF) analysis of whole-mount PLNs, rather than conventional flow cytometry (13). In brief, after optical clearing of entire PLNs of recipient mice, we optically sectioned large 3-D volumes using 2PM scanning to detect rare adoptively transferred cells, in addition to their precise localization with regard to the HEV network (Figure S3C in Supplementary Material). Using this approach, we were able to identify adoptively transferred cell populations and compare their relative frequency with one of the input population. We found that, despite comparable expression of the adhesion molecule L-selectin on both cell populations (not shown) and increased LFA-1 expression on KJ1–26^+^ CD4^+^ T cells (Figure 1F), DO11.10 CD4^+^ T cells were underrepresented in PLNs as compared to their input frequency (Figure S3D in Supplementary Material). While this may have been partially due to lower CCR7 levels on activated DO11.10 CD4^+^ T cells on day 3 p.i., these data are consistent with the generally reduced *in vitro* chemotactic responsiveness of recently activated CD4^+^ T cells to chemokines (Figure 3).

### Transient increase in pStathmin levels on *In Vivo*-activated DO11.10 CD4^+^ T cells

**Figure 4:**
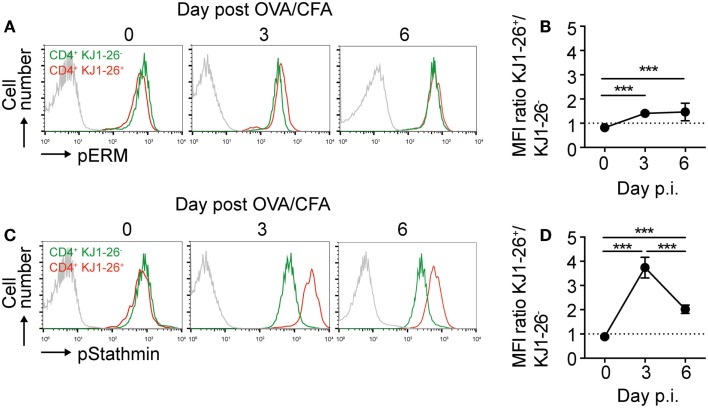
**Dynamic regulation of pERM and pStathmin levels in DO11.10 CD4^+^ T cells**. **(A)** Representative flow cytometry plots showing intracellular pERM levels on endogenous CD4^+^ versus KJ1–26^+^ DO11.10 CD4^+^ T cells on days 0, 3, and 6 post OVA/CFA immunization. The gray line represents secondary Ab only. **(B)** Ratio of pERM levels on endogenous CD4^+^ versus KJ1–26^+^ DO11.10 CD4^+^ T cells. **(C)** Representative flow cytometry plots showing intracellular pStathmin levels on endogenous CD4^+^ versus KJ1–26^+^ DO11.10 CD4^+^ T cells on days 0, 3, and 6 post OVA/CFA immunization. The gray line represents secondary Ab only. **(D)** Ratio of pStathmin levels on endogenous CD4^+^ versus KJ1–26^+^ DO11.10 CD4^+^ T cells. Data in **(A,C)** are representative of two independent experiments, while data in **(B,D)** are pooled from six mice in two independent experiments and shown as mean ± SD. Data were analyzed using ANOVA with Tukey’s multiple comparison test. ***p < 0.001.

Since chemokine responsiveness of recently activated DO11.10 CD4^+^ T cell did not match well with chemokine receptor surface levels, we hypothesized that activation-induced intracellular signaling pathways resulted in lower intrinsic cell motility. *In vitro* TCR activation of human T cells leads to ERM dephosphorylation and stathmin phosphorylation, which cause decreased T cell motility owing to decreased uropod formation and increased microtubule network stability (25). When we examined the phosphorylation status of these molecules on day 0, 3, and 6 post OVA/CFA immunization, we found a minor 1.5-fold sustained increase in pERM levels in DO11.10 CD4^+^ T cells as compared to endogenous CD4^+^ T cells (Figures 4A,B; Figure S1E in Supplementary Material). By contrast, we observed a transient and substantial fourfold increase in pStathmin levels in DO11.10 CD4^+^ T cells on day 3 p.i., which decreased by day 6 p.i. (Figures 4C,D). Of note, pStathmin levels remained elevated for at least 2 h after T cell isolation (not shown), suggesting that high pStathmin levels were maintained in the absence of continued TCR stimulation.

### Microtubule network destabilization, integrin blocking, or PKC inhibition does not restore the migratory capacity on recently activated T cells

**Figure 5:**
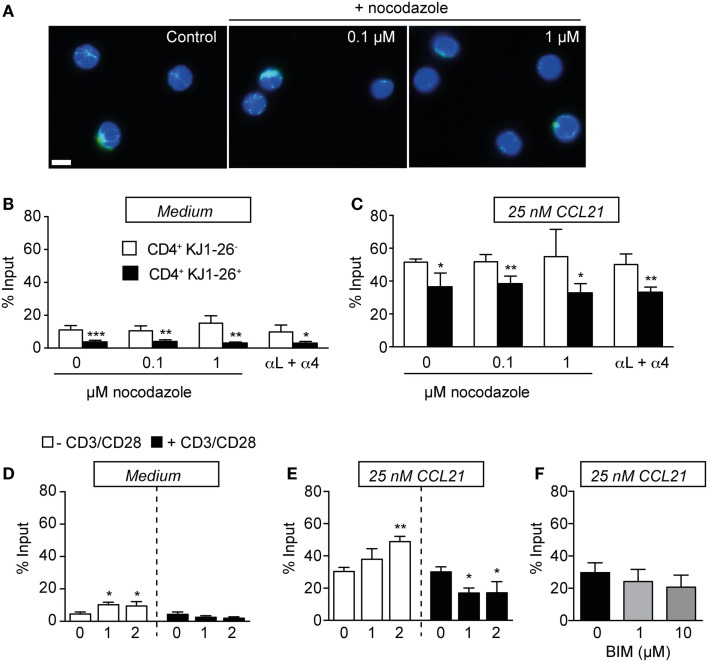
**Activation-induced decrease in T cell motility is maintained in the presence of microtubule destabilization agents, integrin blocking, and PKC inhibition**. **(A)** Immunofluorescent analysis of microtubule networks (green) of CD4^+^ T cells in the presence or absence of nocodazole. Nuclei are labeled with DAPI (blue). Scale bar, 5 μm. **(B)** Chemokinesis of endogenous CD4^+^ versus KJ1–26^+^ DO11.10 CD4^+^ T cells on day 3 after OVA/CFA immunization in the presence of nocodazole or integrin-blocking mAbs. **(C)** Chemotaxis to 25 nM CCL21. **(D)** Chemokinesis of day 2 *in vitro*-activated lymphocytes mixed 1:20 with non-activated lymphocytes. **(E)** Chemotaxis of *in vitro*-activated lymphocytes. **(F)** Chemotaxis of day 2 *in vitro*-activated lymphocytes to 25 nM CCL21 in the presence of the PKC inhibitor BIM. No significant differences were detected. Chemokinetic migration was subtracted in **(C,E,F)** to focus on chemokine-induced motility. Bars represent mean ± SD of % input. Data in **(B,C)** are pooled from two independent experiments with five mice total and were analyzed using an unpaired Student’s *t*-test. Data in **(D–F)** are from one to three independent experiments with a total of four mice and were analyzed using ANOVA and Sidak’s multiple comparison tests. *p < 0.05; **p < 0.01; ***p < 0.001.

It is well established that pStathmin levels positively correlate with microtubular network stability (34, 35). We therefore examined whether disrupting microtubule stability using nocodazole reverses the low *in vitro* motility of activated CD4^+^ T cells. We isolated PLNs 3 days after OVA/CFA immunization and pre-incubated single cell suspensions with increasing concentrations of nocodazole prior to and during chemotaxis. Using immunofluorescence, we confirmed that increasing nocodazole treatment disrupted the lymphocyte microtubule network (Figure 5A). By contrast, nocodazole treatment at 0.1 and 1 μM did not alter chemokinetic motility and chemotaxis to CCL21 of neither endogenous nor KJ1–26^+^ CD4^+^ T cells (Figures 5B,C). We made similar observations with both lower (0.01 μM) and increased (10 μM) nocodazole concentrations (not shown). Next, we investigated whether increased integrin adhesiveness causes cell aggregation, thereby decreasing *in vitro* migration. Yet, similar to nocodazole treatment, blocking α4 and αL integrins did not rescue the reduced KJ1–26^+^ CD4^+^ T cell migration in the absence or presence of CCL21 (Figures 5B,C).

Finally, we examined the role of a potential TCR-driven PKC-mediated phosphorylation leading to a loss of chemokine receptor signaling through receptor desensitization. To obtain activated CD4^+^ T cells in large enough numbers for Western blot analysis, we performed *in vitro* activation experiments using anti-CD3/28 mAb stimulation. Owing to lack of appropriate tools, we were unable to directly assess CCR7 phosphorylation levels in activated T cells (not shown). Yet, we confirmed that in both freshly isolated and day 2 *in vitro*-activated lymphocyte suspensions, conventional, novel, and atypical PKC isoforms were expressed and phosphorylated. In particular, activated lymphocytes showed increased phosphorylation of PKCζ/λ and PKCμ (Figure S4 in Supplementary Material). Furthermore, when we mixed freshly isolated and day 2 *in vitro*-activated lymphocyte suspensions in a ratio similar to *in vivo*-derived suspensions, we were able to recapitulate decreased chemotaxis of activated T cells to 25 nM CCL21 (Figures 5D,E). Yet, preincubation of day 2 *in vitro*-activated lymphocyte suspensions with the pan-PKC inhibitor BIM did not rescue the decreased chemotactic motility (Figure 5F). Similar results were obtained when we performed migration assays to 50 nM CCL21 (not shown). Taken together, these functional assays argue against a role for microtubular network stability, integrin activity, or PKC activity during the motility shutdown experienced by recently activated CD4^+^ T cells.

## Discussion

TCR-driven CD4^+^ T cell activation imprints substantial changes in gene transcriptional programs, which result in cell proliferation and altered expression of adhesion and chemoattractant receptors. How these changes influence intrinsic CD4^+^ T cell motility and responsiveness to chemoattractants has not been examined in detail to date. In this study, we show that recently activated CD4^+^ T cells show a temporal unresponsiveness to spontaneous and chemokine-induced motility *in vitro* and *in vivo*, which was uncoupled from integrin and chemokine receptor levels, PKC activity, or microtubular network stability. This transient low-motility pattern was particularly noticeable under conditions of low-chemotactic gradient strength, since increasing concentrations of CCL21 were able to partially rescue this defect.

Two-photon microscopy studies have uncovered a multistep model for T cell–DC interactions, which are characterized by a gradual decrease in T cell motility until reaching a threshold for complete arrest and long-term interactions with cognate pMHC-bearing DCs. Finally, after detachment from DCs and commitment to cell proliferation, CD4^+^ T cells resume motility, albeit at lower speeds than the ones shown by unstimulated naïve T cells (4, 6). Shortly after activation, T cells increase CD69 expression and downregulate S1P_1_, which contributes to lymphocyte sequestering in lymphoid tissue (36). Our data provide evidence for a cell-intrinsic mechanism regulating basal and chemokine-triggered motility in proliferating CD4^+^ T cells, which is largely independent of the surface expression levels of CCR7 or CXCR4 and may also contribute to lymphocyte sequestering in lymphoid tissue. Thus, we observed reduced *in vitro* T cell motility as early as 1–2 days p.i., when chemokine receptor levels were unchanged. In contrast to CD8^+^ T cells, which undergo an “autopilot” program of multiple cell divisions after a short DC-mediated stimulus (37), CD4^+^ T cells require continuous signaling for full proliferation (38, 39). Thus, reduced motility shortly after activation may constitute an additional mechanism to ensure sufficient PLN dwelling time to engage in productive interactions with late-arriving DCs or prolonged signal integration by exposure to inflammatory cytokines. In line with this hypothesis, activated CD4^+^ T cells readily engage with late-arriving DCs leading to increased activation (10). Thus, a temporal shutdown of CD4^+^ T cell responsiveness to chemoattractants may ensure prolonged dwelling time for full signal integration. At later time points, reduced CCR7 levels during inflammation correlate with increased S1P_1_ levels, which facilitate egress of recently activated lymphocytes (16). An interesting aspect of our analysis is the similar migration of endogenous CCR7^high^ versus CCR7^low^ DO11.10 CD4^+^ T cells to CCL19 or CCL21 after day 6 p.i., although the latter population contained CXCR5^high^ CD4^+^ T follicular helper (T_FH_) cells residing in B cell follicles. These data suggest that chemokine receptor levels on T cells do not necessarily correspond with *in vitro* migratory capacity, similar to what has been described in B cells (40). The recovery of robust migration in activated CD4^+^ T cells starting on day 6 p.i. is in line with the recently described high motility of T_FH_ within germinal centers, as well as their ability to move between follicles (41).

Two-photon microscopy studies have demonstrated that after detachment from DCs, recently activated CD4^+^ T cells display a confined swarming behavior in proximity to DC clusters (6), and *in vitro*-activated T cells typically form LFA-1-dependent clusters. We hypothesized that increased LFA-1-mediated adhesion to its ligands ICAM-1 and -2 expressed on other hematopoietic cells may cause these cells to migrate less efficiently in our *in vitro* assays. Yet, mAb blocking did not recover the low motility of activated CD4^+^ T cells. Taken together, our data show that extracellular factors are unlikely to be involved in decreased DO11.10 CD4^+^ T cell motility.

What are the TCR-triggered intracellular signaling events causing low-intrinsic cell motility? On day 3 p.i., we observed a TCR-driven peak in pStathmin level in proliferating CD4^+^ T cells, pointing to an active microtubule network required for ongoing cell divisions. Pharmacological disruption of the microtubular network by nocodazole or other drugs interferes with LFA-1-dependent *in vitro* motility of human T cell lines (42, 43), as well as with CCL21-triggered LFA-1 adhesiveness of lymphocytes under flow (44). By contrast, nocodazole had previously been used to enhance integrin-independent migration of a CD4^+^ T cell line through 3 μm pores by relaxing the microtubular network (45). Here, we found no effect of microtubule network disruption on shear-free LFA-1-independent migration of mouse T cells, similar to previous assays using primary human T cells performed in the absence of LFA-1 ligands (44, 46). In support of this, a recent publication found that murine T cells in cell cycle accumulated and migrated efficiently within infected tissue (47), indicating that cell cycling *per se* does not generate an overall lack of motility.

Although PKC has been reported to mediate CCR7 phosphorylation and desensitization (27), we found that PKC inhibition did not rescue the defective migration of recently activated CD4^+^ T cells. Owing to a lack of appropriate mAbs, we were unable to examine CCR7 phosphorylation levels in the presence or absence of PKC activity (not shown). We can therefore not exclude a regulatory function of PKC function on chemokine receptors in T cell. However, the fact that chemokinetic motility of activated T cells was also decreased supports a general migratory defect in these cells. In this context, a recent publication demonstrated that coupling of the plasma membrane to the underlying actin cytoskeleton by the adaptor molecule myosin 1g (myo1g) determines membrane tension (48). Since myo1g expression is transiently augmented after TCR activation (http://www.immgen.org), this may contribute to increased membrane tension and as a consequence, a general reduction in cell motility. Future experiments need to be performed to address the molecular mechanisms underlying temporal unresponsiveness.

In sum, we have used a carefully calibrated *in vivo* inflammation system in combination with flow cytometry and *in vitro* chemotaxis to investigate the migratory properties of TCR-stimulated CD4^+^ T cells and its relation with chemokine receptors, integrins, PKC activity, and the microtubular network. Although we were unable to reverse the decreased migration through pharmacological intervention, we hypothesize that the intrinsic low-cell motility may constitute a mechanism that contributes to sequestering of activated CD4^+^ T cells in reactive PLNs, similar to increased surface CD69 and decreased S1P_1_ mRNA expression (36).

## Author Contributions

MA, MH, DL, and JS designed and performed experiments, discussed data, and wrote the manuscript.

## Conflict of Interest Statement

The authors declare that the research was conducted in the absence of any commercial or financial relationships that could be construed as a potential conflict of interest.

## Supplementary Material

The Supplementary Material for this article can be found online at http://journal.frontiersin.org/article/10.3389/fimmu.2015.00297/abstract
