# Supplementary material for: In vivo TCR Signaling in CD4+ T Cells Imprints a Cell-Intrinsic, Transient Low-Motility Pattern Independent of Chemokine Receptor Expression Levels, or Microtubular Network, Integrin, and Protein Kinase C Activity
Source: Front Immunol. 2015 Jun 8;6:297. doi: 10.3389/fimmu.2015.00297 (PMC4459086; doi:10.3389/fimmu.2015.00297)
Supplement: Supplementary file 1 [file Image_1.PDF]

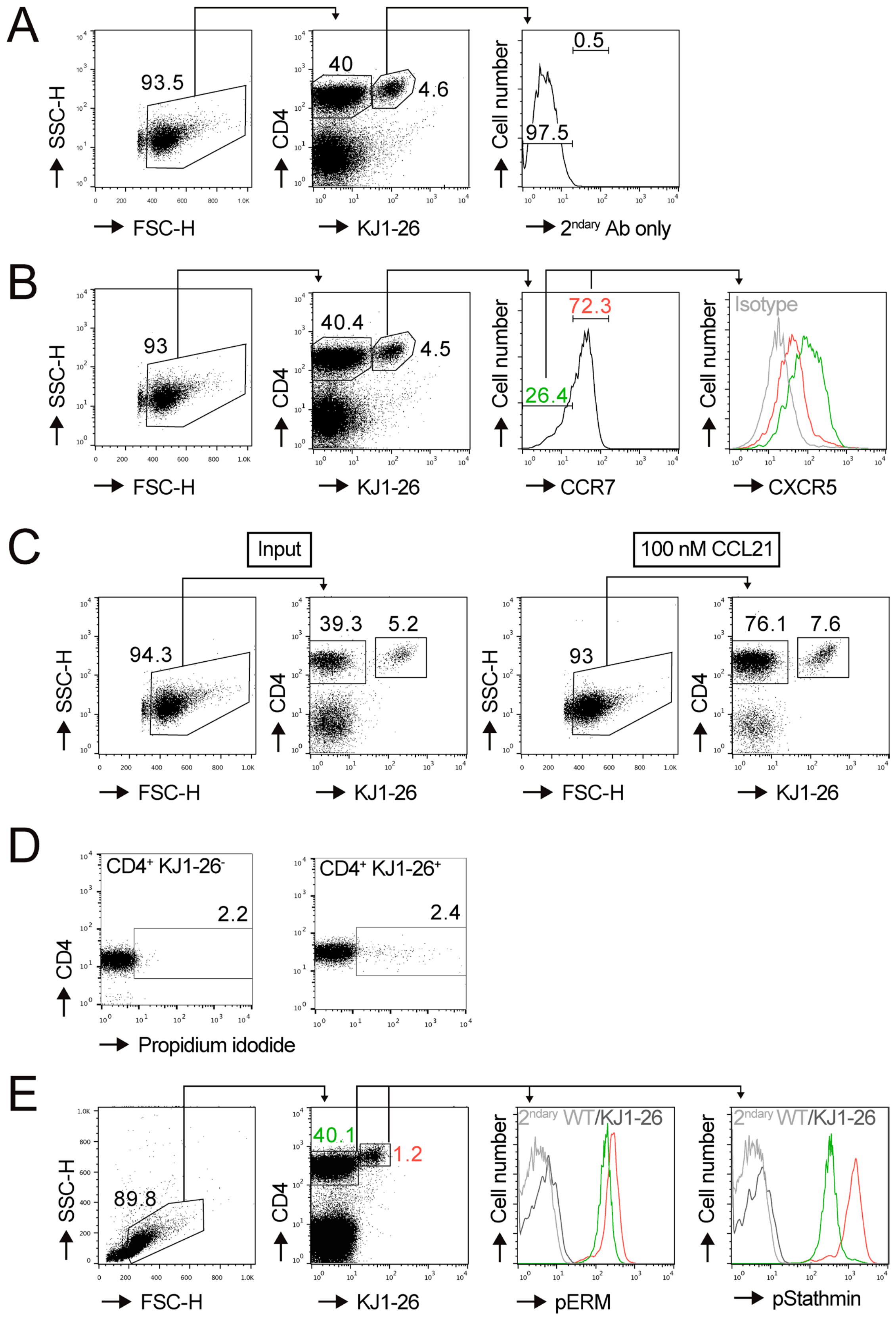

Supplemental Figure 1 | Gating strategies for flow cytometry analysis. **A**. Gating strategy to identify CD4+ KJ1-26-positive and negative populations. The right panel shows staining with secondary anti-human Fc only. **B**. As in **A**, with addition of CCL19-Ig for recognition of CCR7 and anti-CXCR5 mAb for CXCR5 labeling. **C**. Gating strategy for input and migrated lymphocytes in chemotaxis experiments. **D**. Propidium iodide staining of sorted CD4+ KJ1-26- and KJ1-26+ T cells (see Supplemental Figure 3B) after 2 h incubation at 37°C, 5% CO2. **E**. Gating strategy for pERM- and pStathmin labeling. Grey histograms show staining of secondary mAbs only. Numbers in A-E indicate percentages in gated regions. For all flow cytometry strategies, the original input without pregating except the FSC-H threshold is shown on the left-hand dotplot.
