# Supplementary material for: In vivo TCR Signaling in CD4+ T Cells Imprints a Cell-Intrinsic, Transient Low-Motility Pattern Independent of Chemokine Receptor Expression Levels, or Microtubular Network, Integrin, and Protein Kinase C Activity
Source: Front Immunol. 2015 Jun 8;6:297. doi: 10.3389/fimmu.2015.00297 (PMC4459086; doi:10.3389/fimmu.2015.00297)
Supplement: Supplementary file 2 [file Image_2.PDF]

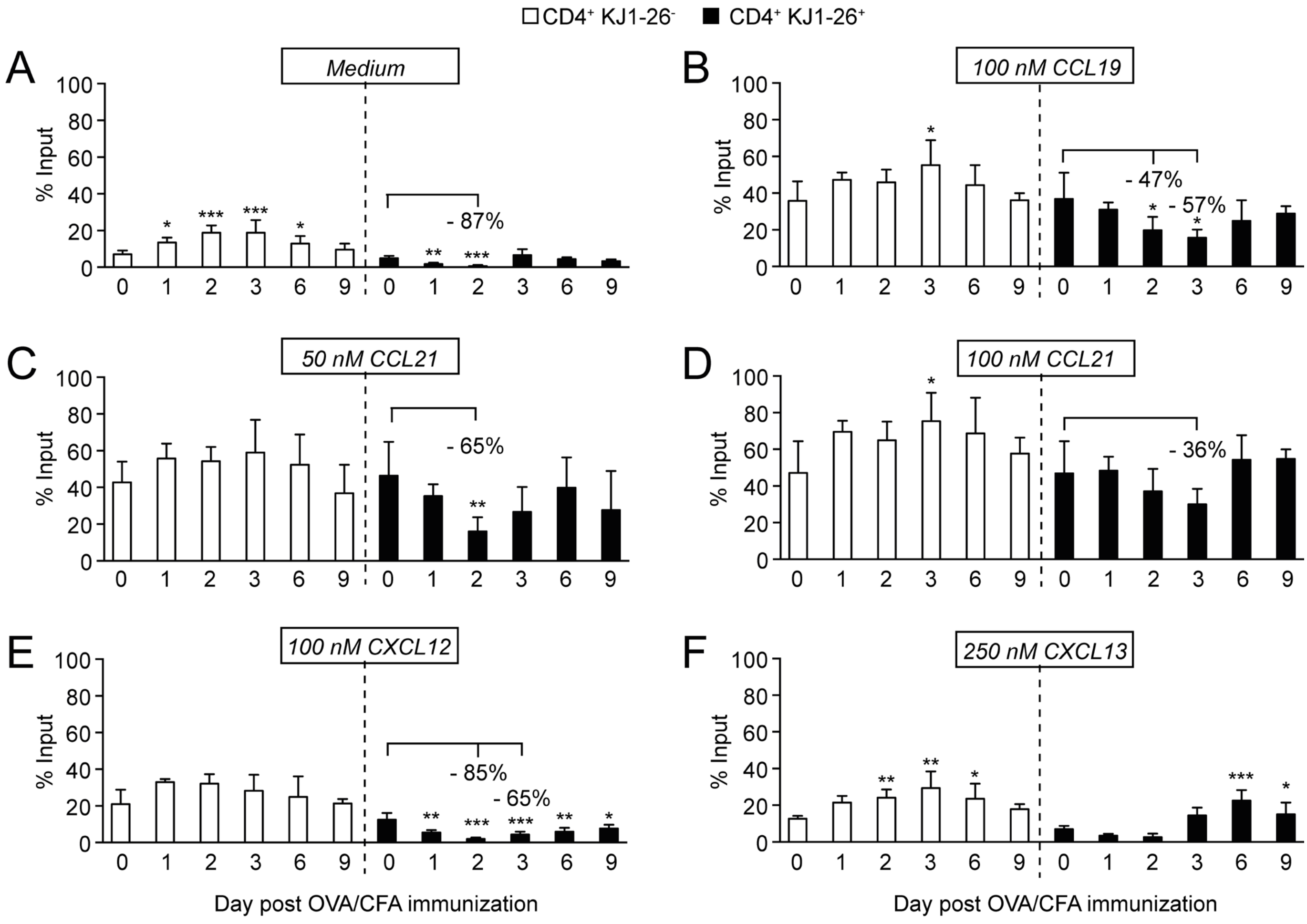

Supplemental Figure 2 | In vitro migration of endogenous and DO11.10 CD4+ T cells during OVA/CFA immunization without background (medium-induced migration) subtraction. A. In vitro chemokinesis of endogenous CD4+ T cells (white bars) and KJ1-26+ DO11.10 CD4+ T cells (black bars) in the absence of chemokine after OVA/CFA immunization. This panel is identical to Figure 3A and is included here for comparison with B-F. **B.** Chemotaxis to 100 nM CCL19. **C**. Chemotaxis to 50 nM CCL21. **D**. Chemotaxis to 100 nM CCL21. **E**. Chemotaxis to 100 nM CXCL12. **F**. Chemotaxis to 250 nM CXCL13. Bars represent mean ± SD of % input and were pooled from 3-4 independent experiments combining 3-8 mice/time point. Statistical analysis was performed separately for endogenous CD4+ T cells and KJ1-26+ DO11.10 CD4+ T cells using ANOVA test against "Day 0" values (= control migration) followed by Dunnett's multiple comparison test. * = $p < 0.05$; ** = $p < 0.01$; *** = $p < 0.001$.
