# Supplementary material for: In vivo TCR Signaling in CD4+ T Cells Imprints a Cell-Intrinsic, Transient Low-Motility Pattern Independent of Chemokine Receptor Expression Levels, or Microtubular Network, Integrin, and Protein Kinase C Activity
Source: Front Immunol. 2015 Jun 8;6:297. doi: 10.3389/fimmu.2015.00297 (PMC4459086; doi:10.3389/fimmu.2015.00297)
Supplement: Supplementary file 3 [file Image_3.PDF]

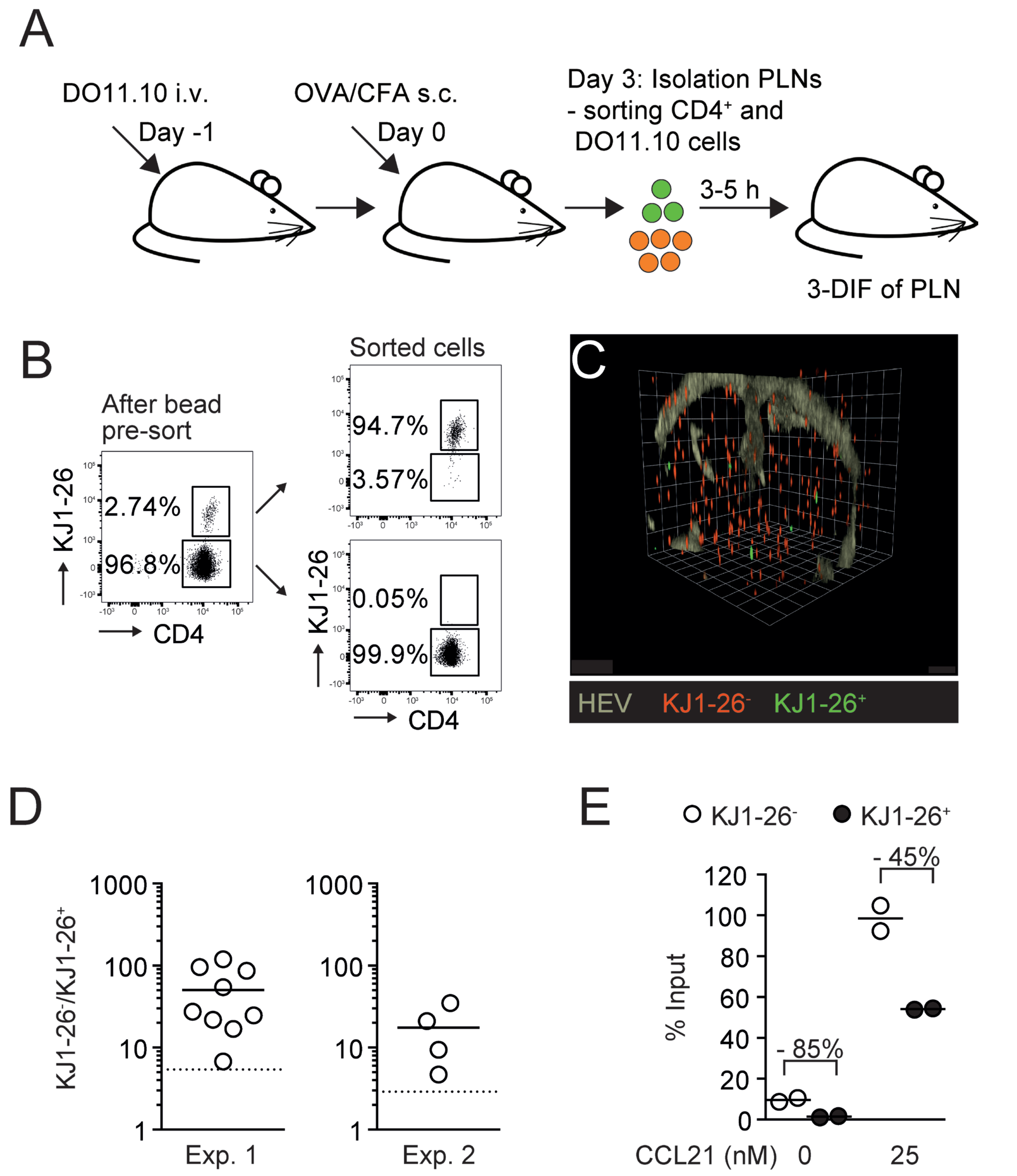


**Supplemental Figure 3 | Reduced in vivo homing capacity of DO11.10 CD4+ T cells on day 3 post OVA/CFA immunization. A.** Outline of homing experiment. **B**. Representative flow cytometry plots depicting sorting of endogenous and DO11.10 CD4+ T cells. **C.** Representative 3-DIF image identifying adoptively transferred KJ1-26- (red) and KJ1-26+ (green) CD4+ T cells and the HEV network (brown). One single grid length corresponds to 40 μm. **D**. Ratio of adoptively transferred KJ1-26- and KJ1-26+ CD4+ T cells as analyzed by 3-DIF in two independent experiments (Exp. 1 and 2). Each dot in the "PLN" column represents the KJ1-26-/KJ1-26+ ratio in individual PLNs after homing. The line indicates the mean, while the dotted line represents the input ratio. **E.** In vitro chemotaxis of flow-cytometry-sorted KJ1-26+ and KJ1-26- CD4+ T cells (2.5 x 105/well, 2 h at 37°C) isolated on day 3 p.i. to medium and 25 nM CCL21. Each dot represents a single well from one experiment.
