# Supplementary material for: In vivo TCR Signaling in CD4+ T Cells Imprints a Cell-Intrinsic, Transient Low-Motility Pattern Independent of Chemokine Receptor Expression Levels, or Microtubular Network, Integrin, and Protein Kinase C Activity
Source: Front Immunol. 2015 Jun 8;6:297. doi: 10.3389/fimmu.2015.00297 (PMC4459086; doi:10.3389/fimmu.2015.00297)
Supplement: Supplementary file 4 [file Image_4.PDF]

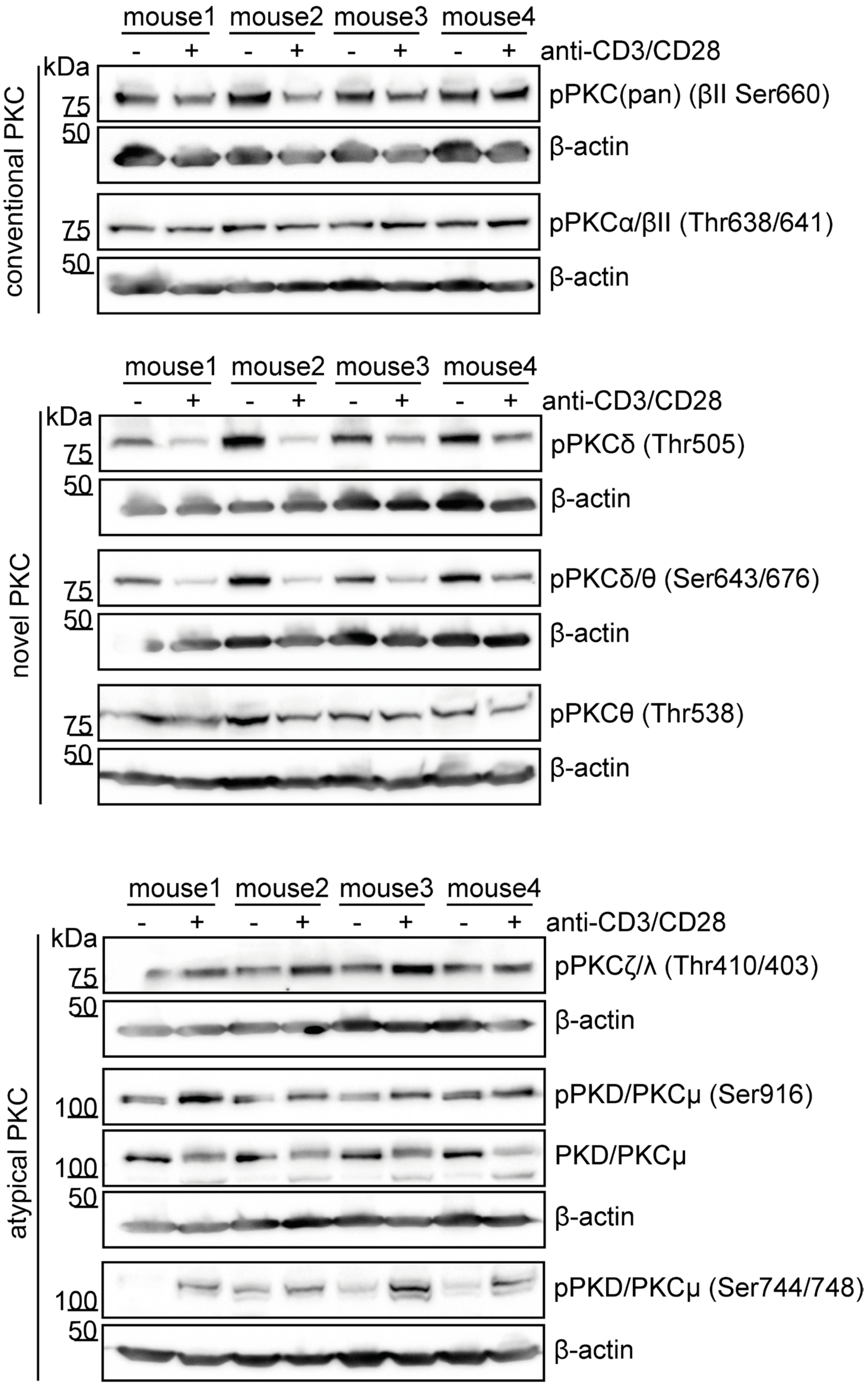

Supplemental Figure 4 | Western blot analysis of conventional, novel and atypical PKC expression and phosphorylation in freshly isolated and day 2 in vitro activated splenocytes. 30 µg protein were loaded per lane.
